# Nanotechnology Meets Oncology: A Perspective on the Role of the Personalized Nanoparticle-Protein Corona in the Development of Technologies for Pancreatic Cancer Detection

**DOI:** 10.3390/ijms231810591

**Published:** 2022-09-13

**Authors:** Damiano Caputo, Erica Quagliarini, Daniela Pozzi, Giulio Caracciolo

**Affiliations:** 1 Department of Surgery, University Campus Bio-Medico di Roma, Via Alvaro del Portillo 200, 00128 Rome, Italy; 2 NanoDelivery Lab, Department of Molecular Medicine, Sapienza University of Rome, Viale Regina Elena 291, 00161 Rome, Italy

**Keywords:** nanotechnology, nanoparticles, early detection, oncology, cancer, pancreatic cancer, pancreatic ductal adenocarcinoma

## Abstract

In recent years nanotechnology has opened exciting opportunities in the struggle against cancer. In 2007 Dawson and coworkers demonstrated that nanomaterials exposed to biological fluids are coated with plasma proteins that form the so-called “protein corona”. A few years later our joint research team made of physicists, chemists, biotechnologists, surgeons, oncologists, and bioinformaticians introduced the concept of “personalized protein corona” and demonstrated that it is unique for each human condition. This concept paved the way for the development of nano-enabled blood (NEB) tests for the diagnosis of pancreatic ductal adenocarcinoma (PDAC). These studies gave an impetus to serious work in the field that came to maturity in the late 2010s. In this special issue, we provide the reader with a comprehensive overview of the most significant discoveries of our research team in the field of PDAC detection. We focus on the main achievements with an emphasis on the fundamental aspects of this arena and how they shaped the integration of different scientific backgrounds towards the development of advanced diagnostic technologies. We conclude the review by outlining future perspectives and opportunities to transform the NEB tests into a reliable clinical diagnostic technology for early diagnosis, follow-up, and management of PDAC patients.

## 1. Introduction

Increased exploitation of nanotechnology in biomedical applications has led to significant progress in the field of cancer diagnosis, mirroring the potential of nanostructured biomaterials in personalized medicine [1]. In recent years, the arena of bio-nano interactions has given hope for new perspectives on cancer detection [2]. In the early history of this field, pioneering investigations by Dawson and coworkers clarified that nanoparticles (NPs) immersed in biological fluids are coated with a protein-enriched layer that was termed the “protein corona” (PC) [3]. In the scientific community, many efforts have been made to understand the principles that regulate the formation, structure, and equilibrium composition of the PC. The practically endless number of combinations between NPs with different chemical-physical properties and biological fluids (e.g., blood, saliva, gastric fluids, interstitial fluids) [4], made rationalizing the results a difficult task. The scenario was also complicated by our understanding that the PC is affected by environmental factors such as exposure time, temperature, and shear stress. Looking back over the past 15 years, we highlight the fact that the PC arises from a complex interplay of the NPs’ physical-chemical properties, the protein source, and the environmental factors. Many review papers have examined these aspects in detail [5,6,7,8] and, therefore, we refer the reader to these contributions for more in-depth information. We want to focus on the fact that the protein corona gives the NPs a new identity, referred to as “biological identity”, which drastically affects its performance in a physiological environment (e.g., biodistribution, toxicity, off-target accumulation, targeted delivery, etc.) [9]. Furthermore, the PC may contain information about a patient’s proteome, and decoding this code has become a priority in biomedical research. After a decade of research, we are now aware that the PC does not merely reflect the composition of the human proteome but preferentially includes plasma proteins with a high affinity for particle surface [10]. This finding had immediate implications in the research of tumor biomarkers. In this context, it was revealed that the nanoparticle-PC is personalized [11], i.e., it is unique for each individual and each human condition. The characterization of the PC may therefore reveal subtle changes in protein biomarkers that are too low to be detected by routine blood tests. Since 2012, our research groups located at the NanoDelivery Lab of the Molecular Medicine Department of the Sapienza University of Rome and the General Surgery Unit of the Fondazione Policlinico Universitario Campus-Biomedico di Roma have been working on the design, development, and validation of new technologies for the early cancer detection based on the characterization of the personalized PC. In early 2012, a protocol was approved by the Ethical Committee of the University Campus-Biomedico (UCBM) di Roma to perform these studies. Our research would not have been possible without the genuine contribution of our coworkers led by Prof. Laganà at the Chemistry Department of the Sapienza University of Rome and the generous financial support of the Italian Minister of Health and the AIRC foundation. In 2014, we introduced the concept of personalized PC for pancreatic ductal adenocarcinoma (PDAC) [12,13]. We focused on this malignancy due to the a lack of effective tests for early detection of PDAC mostly because of the asymptomatic nature of this tumor at the early stages. The only PDAC biomarker approved by the US Food and Drug Administration (FDA) is carbohydrate antigen (CA) 19.9, but it exhibits non-adequate sensitivity (60–70%) and specificity (70–85%) values [14]. In a couple of pilot investigations, we demonstrated that the protein corona from PDAC patients had different charge [12] and composition [13] than those from healthy subjects and other cancer types such as breast and gastric cancer. These studies gave impetus to more serious work in the field. A result of the introduction of the personalized protein corona was the development of a new field setting the foundations and defining the principles of protein corona-based tests for PDAC detection. Due to a fruitful interaction between physicists, chemists, biotechnologists, surgeons, oncologists, and bioinformaticians, the field came to maturity in the late 2010s. According to the topic of this Special Issue, we will provide the reader with a comprehensive overview of the most significant discoveries of our joint research team in the field of PDAC detection. First, we will illustrate the main results that we obtained using PC characterization by liquid chromatography tandem mass spectrometry (LC-MS/MS) (reviewed in [15]). We will then clarify how the limitations highlighted by these studies prompted us to characterize PC using a combination of benchtop techniques such as one-dimensional sodium dodecyl-sulfate polyacrylamide gel electrophoresis (1D SDS-PAGE), dynamic light scattering (DLS), and micro-electrophoresis (ME). The result of these efforts has led us to design, validate, and finally patent the nanoparticle-enabled blood (NEB) tests for cancer detection [16]. The accumulated experience allowed us to identify some weaknesses of this technology. Among these, the isolation of the PC from the particle surface is undoubtedly the most critical point, as it requires a laborious experimental procedure that can compromise the reproducibility of the experimental data. Therefore, the following efforts were focused on the development of NEB tests based on the indirect characterization of the protein corona, i.e., without altering the nanoparticle-PC organization as it occurs in Magneto–Archimedes levitation (MagLev) experiments [17,18].

## 2. Personalized Protein Corona: The Missing Piece for Early-Stage Cancer Detection

**Figure 1:**
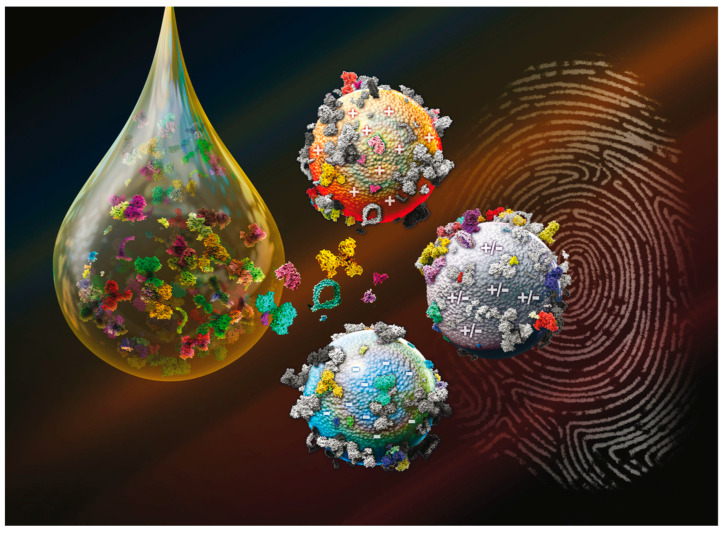
Upon exposure to human plasma proteins, nanoparticles (NPs) are coated by a personalized protein corona. Using a cross-reactive sensor array made of three liposomal formulations, fingerprints for five different cancer types were identified. Adapted from Caracciolo et al. [30].

Most tumors are diagnosed too late for curative treatments. In addition, modern systemic and targeted drugs for treating advanced cancer are expensive and often have limited efficacy [19]. Survival rates significantly increase when the cancer is identified at early stages, namely average 5-year survival at an early stage is 91%, while the average 5-year survival at a late stage is 26% [20]. This emphasizes the urgency of developing robust high-throughput platforms to enable the discovery of new molecular biomarkers for early-stage cancer diagnosis aided by artificial intelligence (AI) based techniques [21]. Currently, there are limited non-invasive screening tests, such as prostate-specific antigen (PSA) tests for prostate cancer [22], colonoscopy for colorectal cancer [23], mammograms for breast cancer [24], and low-dose computed tomography (LDCT) scan for lung cancer [25]. Most of them detect only a minute fraction of potential biomarkers due to their extremely low concentration in biofluids. In addition, the “swamping” effect, caused by non-specific highly abundant molecules, limits the diagnostic information that can be obtained. Blood (serum or plasma) is considered the most valuable biofluid for tumor biomarker discovery as it contains the most comprehensive human proteome, including tissue leakage proteins that reflect ongoing disease states. However, the discovery of biomarkers present in the blood for early detection of pancreatic cancer is challenging and it is hampered by the large dynamic concentration range of blood proteins [26]. Previous strategies, such as targeted and untargeted LC-MS/MS, which typically need depletion of abundant proteins and involve labor- and time-consuming complex workflows, have been developed to expand the protein profile. However, a large fraction of the plasma proteome, potential biomarkers, and disease signatures remain unexplored in large-scale proteomics studies. After decades of research aimed at improving the effectiveness and accuracy of cancer diagnostics, studies conducted on bio-nano interactions have proven useful. It was found that the personalization of the PC depends on the physicochemical properties of NPs (e.g., size, shape, surface chemistry, zeta potential, etc.), the protein source [10] (e.g., blood, gastric fluid, interstitial fluids, etc.), and environmental factors (temperature [27], shear stress [28], etc.). This opens a new avenue to sequester low-abundant proteins in a biospecimen ex vivo and thus increases the possibility of detecting cancer biomarkers through downstream analytical workflows. As an example, Hadjidemetriou et al. showed that the PC that forms around liposomes in vivo contained low molecular weight proteins and poorly abundant cancer-specific proteins that could not be detected by conventional proteomics in both experimental disease models and human subjects [29]. This new fascinating approach that looks at the PC in a “personalized” manner, has numerous implications in the field of tumor diagnostics, since the accurate characterization of the PC can predict how the proteome is affected by the tumor and, eventually, how it evolves in time. In a former investigation [30], we developed a cross-reactive sensor array platform with cancer detection capacity made of three liposomal formulations with different surface charges, i.e., cationic 1,2-Dioleoyl-3-trimethylammonium propane (DOTAP), anionic 1,2-Dioleoyl-sn-glycerol-3-phosphoglycerol (DOPG) and the zwitterionic mixture made of dioleoylphosphatidylcholine (DOPC) and cholesterol (hereafter indicated as L1, L2, and L3). We used HP from patients affected by lung cancer, glioblastoma, meningioma, pancreatic ductal adenocarcinoma (PDAC), and myeloma. The coronas were characterized by LC-MS/MS and after accurate statistical analysis, it was found that changes in the corona composition pattern could provide a unique “fingerprint” for each type of cancer (Figure 1).

Final validation on a sample cohort demonstrated that the detection platform could correctly discriminate cancer patients from healthy volunteers with high detection accuracy >99%. In a subsequent proteomic investigation [31], we carried out a comparative analysis of the corona formed on the same three liposomal formulations upon incubation with HP from PDAC patients and healthy subjects. Complement C4-A and C4-B (CO4A and CO4B) were found to be significantly downregulated in all the PDAC coronas, while complement component 3 (CO3), C4b-binding protein (C4BPA), and platelet basic protein (CXCL7) were significantly downregulated in PDAC samples for two out of three liposome formulations. Among the upregulated proteins in PDAC samples, the most significant differences corresponded to fibrinogen gamma chain (FIBG), serum amyloid A-2 (SAA2), and apolipoprotein C-II (APOC2), which were detected with larger relative protein abundances for at least one of the three formulations. Fibrinogen alpha and beta chains (FIBA and FIBB) were more abundant in PDAC coronas for L1 and in “control” coronas for L3. The same trend was detected for apolipoprotein C-III (APOC3), while apolipoprotein E (APOE) and apolipoprotein A-II (APOA2) were more abundant in cancer coronas for L2 and less abundant for L1 and L3, respectively. Motivated by these findings we explored the personalized protein corona of graphene oxide (GO) nanoflakes in the human plasma of pancreatic cancer patients [32]. GO was chosen due to extraordinary physical-chemical properties that prompt researchers to apply it in disease-related diagnostics [33]. In our investigation, we focused on previously unexplored factors (e.g., GO lateral size) that shape the GO-PC in biological media. Notably, protein apolipoprotein A1 (APOA1) was the most enriched plasma protein. That protein was already recognized as a potential biomarker for PDAC [34]. Other representative proteins detected included serum albumin (ALB), hemoglobin (subunit B and A1, HBB and HBA1 respectively), and alpha-1-antitrypsin (SERPINA1). In previous studies, SERPINA1 was identified as a clinically useful biomarker for prognostic or therapeutic purposes in metastatic pancreatic cancer [34,35]. Remarkably, and differently from our previous findings, we found that SERPINA1 was the one protein with a significant PDAC-to-healthy RPA fold-change. Overall, proteomic investigations demonstrated that protein biomarkers associated with pancreatic cancer (and potentially with its progression) could be identified within the protein corona. However, we also clarified that PC characterization by LC-MS/MS is not aligned with the ASSURED (Affordable, Sensitive, Specific, User-friendly, Rapid and robust, Equipment-free, and Deliverable to end-users) criteria stated by the World Health Organization (WHO) for the screening and detection of cancer. Thus, our next challenge was determining the protein corona of pancreatic cancer patients using characterization methods satisfying the ASSURED criteria and additional standards that emerged [36]. These efforts led to the development of nanoparticle-enabled blood (NEB) tests.

## 3. Nanoparticle-Enabled Blood Tests

**Figure 2:**
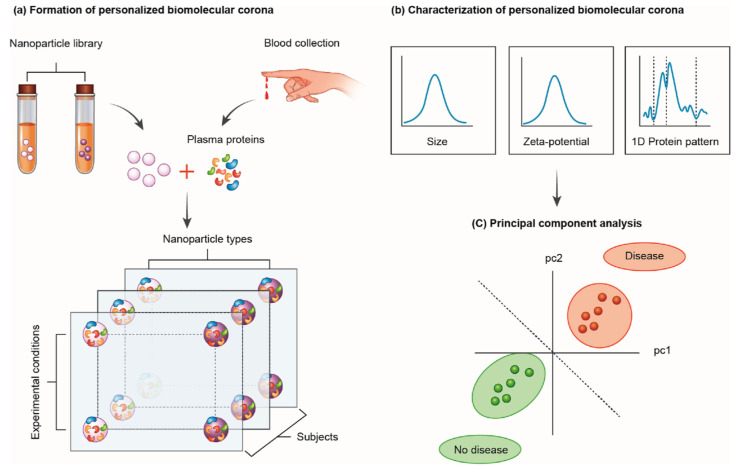
Nanoparticle-enabled blood tests for early cancer detection. (**a**) Nanoparticles are incubated with human plasma collected from cancer patients and healthy subjects. (**b**) A library of nanoparticle-protein complexes is generated by exposing different NP types (e.g., gold NPs, GO nanoflakes, liposomes, lipid NPs, etc.) with different physical-chemical properties (e.g., size, aggregation state, shape, surface charge, etc.) to HP under different experimental conditions such as protein concentration, NP/protein molar ratio, temperature, shear stress, etc. This practice results in the development of a big data set of “personalized protein coronas”. (**c**) For each corona, several physical-chemical parameters are collected.The data set is compacted by principal component analysis (PCA) into a series of new variables (PC1, PC2, etc.). Cancer patients and healthy volunteers give rise to two well-separated clusters in the principal components’ Cartesian plane (“disease” vs. “no disease”). Reprinted with permission from Elsevier from *Nano Today*, 21, 14–17. (2018), M. Papi and G. Caracciolo. Principal component analysis of personalized biomolecular corona data for early disease detection [4].

Inspired by the need to overcome the limitations of LC-MS/MS, the NEB tests have rapidly emerged as a fast, cheap, and useful tool for cancer detection. NEB tests are performed in a step-by-step workflow as schematically represented in Figure 2. These steps include: (i) the collection of clinically relevant body fluids from pancreatic cancer and healthy subjects. To date, only serum and plasma have been used, while other fluids such as saliva are currently under investigation; (ii) the synthesis of a library of NPs with different physical-chemical properties; (iii) the choice of exposure conditions between NPs and body fluids to generate nanoparticle-protein complexes (e.g., temperature, incubation time, shear stress, etc.); (iv) the characterization of size, surface, and protein composition of the complexes; and (v) the statistical analysis of experimental data (e.g., principal component analysis (PCA) followed by linear discriminant analysis (LDA)). The test structure has many degrees of freedom that can affect its prediction ability. First, the physical-chemical properties of NPs can shape the protein corona thus contributing to its personalization. The modulation of the NPs’ surface was essential for the enrichment of low-abundant protein biomarkers leading to the discovery of possible therapeutic targets for ovarian cancer [37]. For the NEB test to work at its best, it is necessary to choose an NP type (e.g., 100 nm spherical gold nanoparticles) that has an affinity for those plasma proteins which are significantly altered in cancer patients. In this way the corona protein will work as a nano-concentrator of those proteins and, consequently, it will be possible to identify a “cancer signature” among the physical-chemical properties of the nanoparticle-protein corona (e.g., size, zeta-potential) or in the composition of the protein corona itself. Since the alterations of the proteome of the cancer patient are not known a priori, it is not possible to choose an NP type with a selective affinity towards them. Therefore, it is essential to screen NP types and exposure conditions such as protein concentration [32], shear stress [38], exposure time [39,40], and temperature [41]. For each corona, several physical-chemical parameters can be determined, such as size, zeta-potential, and protein profiles, by combining dynamic light scattering (size), micro-electrophoresis (zeta-potential), and 1D SDS-PAGE (1D protein patterns). The big data set is compressed by principal component analysis (PCA) into a series of new variables (PC1, PC2, etc.). When the procedure is optimized, cancer patients and healthy volunteers will give rise to two well-separated clusters in the principal components’ Cartesian plane (“disease” vs. “no disease”). Other statistical methods can be used to analyze protein corona data. The receiver operating characteristic (ROC) curve can be also applied to measure the diagnostic accuracy of the NEB test [42].

**Figure 3:**
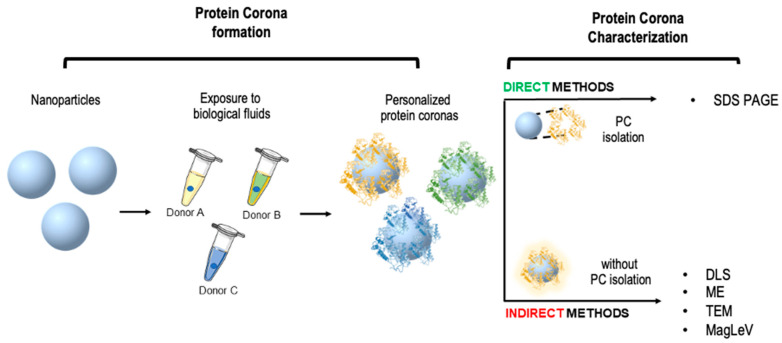
Schematic representation of nanoparticle-enabled blood (NEB) test for personalized PC characterization. Nanoparticles are exposed to biological fluids from different donors giving rise to personalized protein coronas. Direct methods of corona characterization require protein isolation from the particle surface, while indirect methods characterize the whole nanoparticle-corona systems.

In the following, we report and discuss the main detection techniques usually applied for PC characterization in the NEB test protocol. Specifically, we compare analytical tools earing to two different methodological approaches: (i) direct characterization of the PC isolated from the nanoparticle surface and (ii) indirect characterization of the PC which consists of an in-situ evaluation of the NP-PC complexes (Figure 3).

## 4. NEB Tests Based on Direct Characterization of the Personalized Protein Corona

Among direct methods for developing the NEB test, SDS-PAGE has been the most used. When the NEB test is based on SDS-PAGE, the main outcome is not cancer biomarker discovery, as for LC-MS/MS analysis, but the overall evaluation of the protein profiles. This means that the differentiation between cancer patients and healthy subjects arises from a global change of plasma proteins bound to NPs. In a pilot study [43], we developed a version of the NEB test using DOPG liposomes, HP, DOPG/protein ratio 1:1. (vol/vol), bulk mixing, 20 °C, and 1-h as NP type, protein source, NP/protein ratio, shear stress, temperature, and incubation time. This test variant exhibited an overall prediction ability of 88% and was the proof of concept that characterization of the personalized protein corona may pave the way for the development of powerful diagnostic technologies. Other variants of the NEB test exploited the peculiar properties of gold NPs [44] and graphene oxide (GO) [42], and correctly classified about 80% and 90% of the investigated samples, respectively. Among the nanomaterials used, GO was the most promising one as it generated NEB tests with the highest sensitivity and specificity. The next studies were therefore aimed at optimizing GO-based versions of the test. Interestingly, we demonstrated that protein concentration is a major factor in boosting the prediction ability of the GO-based NEB test [32]. In early 2020 we developed a multiplexed strategy combining the test’s outcomes with hemoglobin levels [45]. This synergistic approach returned high sensitivity (82.4%), and specificity (97.1%). When the sample was restricted to healthy subjects and patients with early-stage PDAC (stage I and II) similar results were obtained in terms of sensitivity and specificity (86.7% and 95.8%, respectively). These findings are encouraging, considering that PDAC patients may have anemia and anemia has been reported to increase the risk of PDAC, but to date, to the best of our knowledge the assay of Hb alone has been widely demonstrated to be burdened by very low specificity for PDAC early detection [46]. The latter result was in agreement with previous findings showing that tools based on the characterization of the personalized PC may improve the accuracy of PDAC clinical staging, identifying among resectable PDACs with potentially better prognosis (i.e., T1 and T2) those at higher risk of occult distant metastases [47]. These results are in line with the recent advances that led to the last edition of the TNM staging system for PDAC, which underlined the importance of the T stage compared to the N stage [48].

Lastly, we mention that the NEB test was employed to detect other cancer types such as lung cancer [49], meningioma [50], and glioblastoma multiforme [51]. Overall, ten years of research have demonstrated that the NEB tests are powerful tools for early cancer detection and may pave the way for the development of novel technologies for the diagnosis and management of PDAC [52]. However, we had to face issues along the road. Among them, isolating the PC is challenging due to potential contamination by loosely bound proteins (i.e., the “soft corona”) and biological NPs present in HP. One of the most widely used methods for PC isolation is centrifugation. While it is not immune from contaminations, the use of charged NPs (e.g., cationic liposomes) keeps contamination issues at a minimum [53]. In addition, the number of steps required for PC isolation may lead to inter-operator variability compromising the reliability of the results. For this reason, in recent years, the indirect characterization of PCs, i.e., without isolating the proteins from nanomaterial surfaces, has rapidly emerged as a promising alternative to reduce the experimental steps without affecting the test’s effectiveness, while enhancing its reproducibility when a large dataset is required. This process will be described in the next section.

## 5. NEB Tests Based on the Indirect Characterization of the Personalized Protein Corona

**Figure 4:**
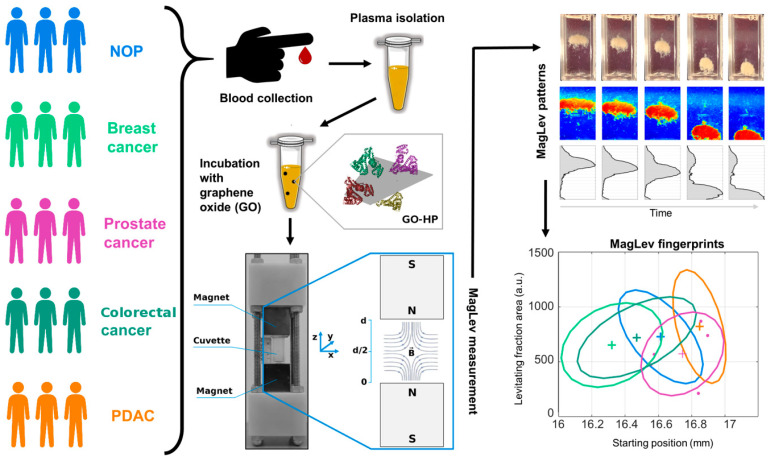
Workflow of the MagLev nanoparticle-enabled blood (NEB) test for cancer detection. Blood from non-oncological patients (NOP) and patients affected by different cancer types is collected. Then human plasma is separated and incubated with NPs such as graphene oxide (GO). NP-HP complexes are injected into a cuvette filled with a paramagnetic medium and inserted into a MagLev device. Levitation patterns are acquired and processed to quantify MagLev profiles. Lastly, fingerprints such as the levitating fraction area and the starting position of the levitating fractions are detected. Reprinted from Quagliarini et al., Nanomaterials 2022, 12, 1397 [18].

Indirect methods for PC characterization look at the NP-protein complex as a unique entity. Size, shape, surface charge, nanostructure, and mass of NP-protein complexes are the principal information acquired through indirect techniques. For instance, the size and surface charge of NPs immersed in biological fluids can be monitored by DLS and ME. In one of our first works [13], we exposed liposomes to HP from healthy and cancer patients (i.e., breast, gastric, and pancreatic cancer) for 1-h and characterized the size and zeta-potential of liposome-protein complexes. The hydrodynamic size of liposome-protein complexes did not exhibit any significant difference between groups, while the zeta-potential of PDAC samples was less negative compared to those of breast, gastric, and healthy ones. However, such changes were not significant enough to prompt us to develop an NEB test based on the zeta-potential of NP-protein complexes. Among indirect approaches, MagLev has recently emerged as an efficient strategy for the indirect characterization of the PC [54]. In a MagLev experiment, diamagnetic objects are injected into a MagLev device filled with a paramagnetic liquid where they migrate in a high-intensity magnetic field (Figure 4). In a typical Maglev-based NEB test, the nanoparticle-protein complexes represent the diamagnetic entity. Since plasma proteins have different densities because of their different MW, shape, and volume, it was shown that once immersed in a paramagnetic liquid and exposed to the high-intensity magnetic field the coronated complexes levitated at specific heights according to their specific own densities [54]. For density measurement purposes, a MagLev system needs to be calibrated by using objects with known densities. However, for diagnostic purposes, accurate calibration can be skipped, and other physical quantities such as the height reached by the levitating fraction, and the time taken to reach the equilibrium state can be used for differentiating the personalized PCs derived from healthy and PDAC-affected plasma donors [17]. In particular, the starting position (i.e., the position reached by levitating fraction at the equilibrium state) and the precipitation speed of coronated-graphene oxide nanosheets were identified as MagLev fingerprints for PDAC, enabling to distinguish 10 PDAC patients from 10 healthy subjects with 80% specificity, 100% sensitivity, and global classification accuracy of 90%. The prediction ability of the method can be optimized by tuning different parameters such as the NP type, the paramagnetic medium, their respective concentration, and the time of profile image acquisition. For instance, the accurate tuning of different conditions may help optimize the Maglev test according to the specific class of disease. In this regard, we demonstrated that our Maglev-based protocol for PDAC detection was not suitable for the classification of other cancer diseases, such as breast, colorectal, and prostate cancer [18]. Variants of the Maglev NEB test may however be developed even for other cancer types by combinations of tunable characteristics, such as the intensity of magnetic field gradient, the paramagnetic medium, the protein source, and the environmental factors (i.e., temperature, incubation time, and shear stress). As an example, we demonstrated that NP-protein complexes generated under shear stress exhibit different levitation profiles with respect to their counterpart produced by bulk mixing [55]. Finally, as MagLev, such as other indirect characterization approaches, are non-destructive technologies, proteins isolated from the devices could be analyzed by analytical techniques, such as LC-MS/MS. To conclude, when confirmed on a larger patient cohort, the MagLev NEB test could be clinically helpful at the first level of investigation to decide whether to perform more invasive tests, or it could be useful for the follow-up of oncological patients after surgery and/or other oncological treatments (e.g., chemotherapy, radiotherapy).

## 6. Conclusions

The research carried out over the past 10 years represents a good example of what science can do when different disciplines meet. Scientists with different backgrounds speak different languages and communication barriers can be difficult obstacles to overcome. In our experience, physicists, chemists, biotechnologists, surgeons, and oncologists have passionately tried to overcome this “potential barrier” and their efforts have paid off. As a first step, we showed that the personalized protein corona of PDAC patients contains a signature associated with the tumor and its stage. Starting from this premise, we elaborated the first NEB test for PDAC detection, and several refined variants have been proposed over time. After a decade of research, the NEB tests represent one of the most promising opportunities for the development of diagnostic technologies for early diagnosis, follow-up, and management of cancer patients [56]. Ongoing research is exploiting the body of knowledge thus gained, and is aimed at overcoming the limitations of the present version of the technology. Among those limitations, the reproducibility of experimental data is the area that needs more attention. The main goal of our current efforts is therefore an accurate standardization of experimental protocols. Similarly to what has already been done for the standardization of the results on the PC characterization [57], we believe that it is essential to standardize all the steps of the diagnostic procedure, from the patients’ enrollment and the blood collection up to the generation of the test output. In this regard, we believe that the choice of inclusion criteria, the procedure for collecting blood from patients, the isolation of plasma, and its storage until use require special attention. The use of low-bind Eppendorf tubes, the accurate purification of the plasma, and the splitting of purified plasma into separate aliquots to be used once and not refrozen are key points that must be rigorously standardized. At an experimental level, the protocol for isolating proteins will have to be uniquely determined since small variations in the centrifugation speed to pellet NP-PC complexes, temperature, and plasma concentration can modify the composition of the personalized PC and, consequently, alter the diagnostic capacity of the NEB test. We also point out that the software for data treatment must be professionalized and coordinated. Finally, we would also like to reiterate that obtaining reliable and reproducible experimental data could benefit from the identification of tumor signatures in a property of the personalized protein corona that does not require the isolation of plasma proteins from the NPs. For example, preliminary experiments carried out in collaboration with the Graz University of Technology have shown that a pancreatic tumor fingerprint may be represented by the nanostructure of the “nanoparticle + protein corona” system detectable by X-ray diffraction. Establishing precise relationships between the nanostructure, the stage of pancreatic cancer, and the effect of surgical and pharmacological treatments are the main goals of our future research. Resolving these questions is essential for transforming the NEB tests into a reliable clinical diagnostic tool.
